# Supplementary material for: Resolving the full spectrum of human genome variation using Linked-Reads
Source: Genome Res. 2019 Apr;29(4):635–45. doi: 10.1101/gr.234443.118 (PMC6442396; doi:10.1101/gr.234443.118)
Supplement: Supplemental Material [file supp_gr.234443.118_Supplemental_longranger-2.2.zip › longranger-2.2.tar.bz2!/lr2.2-public/lib/stan/cmdstan/stan/doc/api/html/structstan_1_1gm_1_1visgen.html]

Stan: stan::gm::visgen Struct Reference


|  |  |
| --- | --- |
|  | Stan  2.5.0  probability, sampling & optimization |


- Main Page
- Related Pages
- Namespaces
- Classes
- Files

- Class List
- Class Index
- Class Hierarchy
- Class Members

All Classes Namespaces Files Functions Variables Typedefs Enumerations Enumerator Friends Macros Pages

- stan
- gm
- visgen

Public Types |
Public Member Functions |
Public Attributes |
List of all members

stan::gm::visgen Struct Reference

generic visitor with output for extension
More...

`#include <generator.hpp>`

Inheritance diagram for stan::gm::visgen:

|  |  |
| --- | --- |
| Public Types | |
| typedef void | result\_type |
|  | |

|  |  |
| --- | --- |
| Public Member Functions | |
|  | visgen (std::ostream &o) |
|  | |

|  |  |
| --- | --- |
| Public Attributes | |
| std::ostream & | o\_ |
|  | |

## Detailed Description

generic visitor with output for extension

Definition at line 64 of file generator.hpp.

## Member Typedef Documentation

typedef void stan::gm::visgen::result\_type

Definition at line 65 of file generator.hpp.

## Constructor & Destructor Documentation

|  |  |  |  |  |  |
| --- | --- | --- | --- | --- | --- |
| stan::gm::visgen::visgen | ( | std::ostream & | *o* | ) |  |

inline

Definition at line 67 of file generator.hpp.

## Member Data Documentation

std::ostream& stan::gm::visgen::o\_

Definition at line 66 of file generator.hpp.

---

The documentation for this struct was generated from the following file:

- src/stan/gm/generator.hpp


---

[ Stan Home Page ]

*© 2011–2014,
Stan Development Team.*
